# The effect of surveillance program for surgical site infection of hepato-biliary-pancreatic surgery for 5 years: a prospective study in a tertiary hospital in Korea

**DOI:** 10.1186/2047-2994-4-S1-P76

**Published:** 2015-06-16

**Authors:** EJ Kim, EH Choi, NJ Kim, BR Oh, JH Lim

**Affiliations:** 1 Infection Control Center, Seoul National University Hospital, Seoul, Republic Of Korea; 2 Internal Medicine, Seoul National University Hospital, Seoul, Republic Of Korea

## Introduction

Surgical site infections (SSIs) constitute one of the most important complications of hepato-biliary-pancreatic surgery [identified as ‘BILI’ in the National Healthcare Safety Network(NHSN) system of the Centers for Disease Control and Prevention], resulting in increased morbidity, mortality and medical expense. Surveillance of SSIs is one of the most effective methods for reducing the incidence.

## Objectives

This study conducted to examine the effect for 5 year surveillance and feedback program of hepato-biliary-pancreatic surgery in a tertiary hospital with 1,700 beds in Korea.

## Methods

This prospective study involved 2,562 patients who were to undergo hepato-biliary-pancreatic surgeries [identified as ‘BILI’ in the NHSN system of the CDC] from 2008 to 2012. For all patients enrolled, the following characteristics were recorded at the time of surgery and during hospitalization: age, sex, ASA score, wound class, emergency or elective surgery, general anesthesia, use of scope, trauma, and risk index score by the NHSN system. SSIs were identified through active concurrent surveillance according to CDC criteria. A confirmed case of SSI was notified to the surgeon and periodically feedback was given to the surgery department. Statistical significant was determined using the Chi-squared test, the Fisher exact test, the t test and a logistic regression analysis of selected variables (P<0.05) was performed to identify risk factors. Statistical calculations were performed by means of the SPSS.

## Results

A total of 2,562 consecutive cases undergoing operations for BILI category and there were 115 cases of SSI for 5 years. The overall SSI rate was 4.49% and SSI rates were significantly reduced than the first year (P=0.012). Superficial incisional SSIs were 19 cases (16.5%), deep incisional SSIs were 11 cases (9.6%) and Organ-space SSIs (intra-abdominal infections) were 85 cases (73.9%). Using a logistic regression model, the following risk factors were age (odds ratio [OR] = 1.045 ; 95% confidence interval [CI], 1.028 - 1.061) and intraoperative time (OR = 1.006 ; 95% CI, 1.005 - 1.008).

## Conclusion

The prospective surveillance and subsequent feedback program were effective for reducing the rates of SSIs of hepato-biliary-pancreatic surgery.

## Disclosure of interest

None declared.

